# Supplementary material for: A Novel Classification Model for Lower-Grade Glioma Patients Based on Pyroptosis-Related Genes
Source: Brain Sci. 2022 May 28;12(6):700. doi: 10.3390/brainsci12060700 (PMC9221219; doi:10.3390/brainsci12060700)
Supplement: Supplementary file 1 [file brainsci-12-00700-s001.zip › brainsci-1677642-supplementary.pdf]

Supplementary Materials:

The results showed that frequent cross-talks among the PRGs were found at the protein level (Figure S1A) and mRNA expression level (Figure S1C). The above data indicated considerable heterogeneities in genetic and expression alterations among LGG and normal brain tissues.

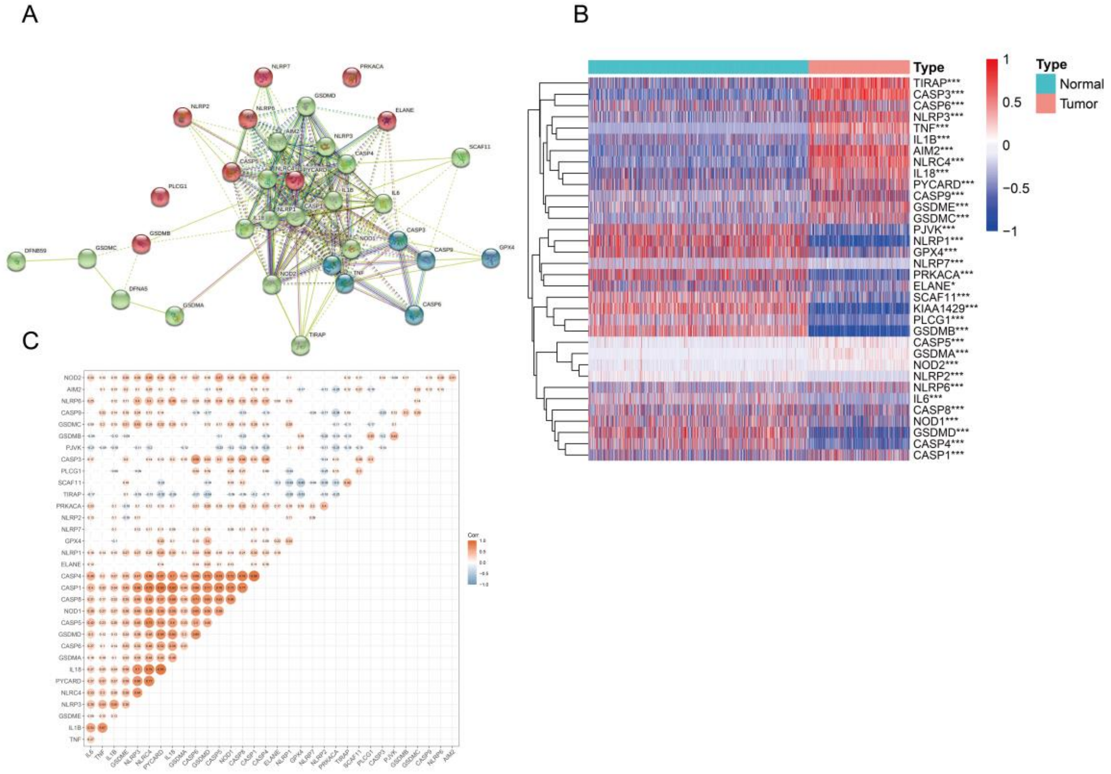

Figure S1. Expression levels of PRGs and their cross-talk. (A) PPI network of PRGs. (B) Heatmap showing the expression levels of PRGs among LGGs and normal brain tissues. (C) Correlation analysis of PRGs.

Table S1. Univariate cox regression analysis of PRGs.

**id**	**HR**	**95LCI**	**95HCI**	***p*-value**
CASP9	0.459118	0.346571	0.608213	5.78E-08
PJVK	0.515016	0.32899	0.806228	0.003709
TIRAP	0.565282	0.338862	0.942992	0.028905
GSDMC	0.753954	0.603173	0.942427	0.013108
TNF	0.852045	0.741511	0.979055	0.023912
GSDMB	0.855963	0.644127	1.137465	0.283677
AIM2	0.873592	0.647656	1.178347	0.376098
GPX4	0.92054	0.515032	1.645324	0.779915
NLRP2	0.927682	0.723168	1.190034	0.554682
IL1B	0.985726	0.881757	1.101953	0.800416
NLRP3	1.04441	0.850695	1.282236	0.678049
ELANE	1.050016	0.638928	1.7256	0.847304
GSDME	1.122868	0.803043	1.570067	0.498066
IL6	1.127872	0.988313	1.287138	0.074175
NOD2	1.172766	0.886162	1.552064	0.264997
NLRP1	1.216858	0.871224	1.699613	0.249603
GSDMA	1.262597	0.983331	1.621174	0.067524

PYCARD	1.328502	1.105496	1.596492	0.002448
NLRC4	1.540594	1.16029	2.045549	0.00281
IL18	1.548205	1.300497	1.843095	8.94E-07
CASP1	1.718926	1.425953	2.072093	1.33E-08
NLRP6	1.76532	1.10206	2.827753	0.018067
SCAF11	1.876782	1.082847	3.252825	0.024857
CASP5	1.96781	1.453363	2.664357	1.20E-05
GSDMD	2.107118	1.729228	2.567587	1.46E-13
CASP4	2.377343	1.898568	2.976855	4.44E-14
NOD1	2.533365	1.879566	3.414585	1.04E-09
CASP6	2.571406	1.999475	3.306934	1.86E-13
PRKACA	2.847789	1.767692	4.587848	1.70E-05
CASP3	2.996859	2.23712	4.01461	1.87E-13
CASP8	3.415454	2.554154	4.567197	1.18E-16
PLCG1	3.519961	2.49644	4.963118	7.03E-13
NLRP7	22.97205	0.714861	738.2058	0.076666

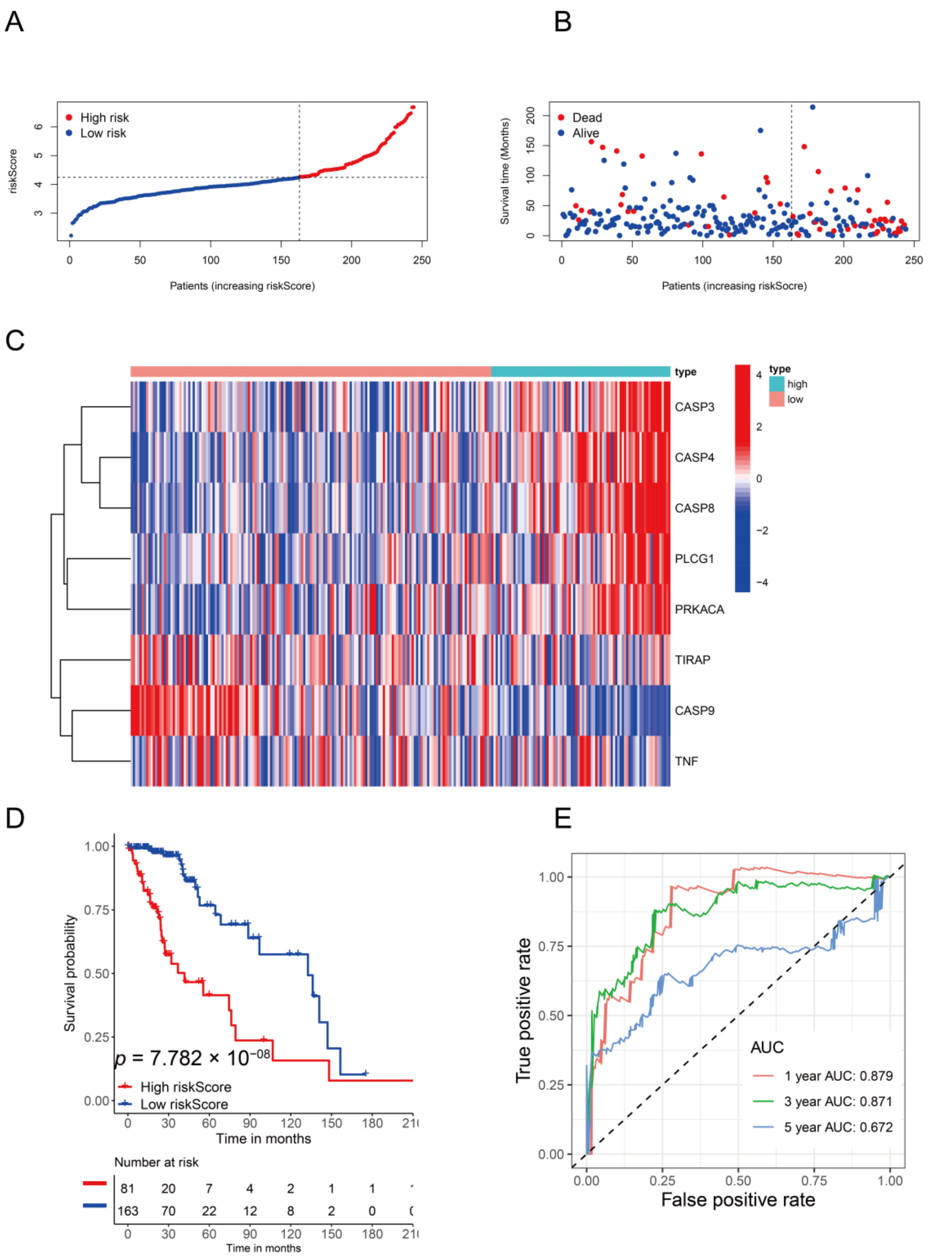

Figure S2. Validation of the PyroScore. (A) Distribution of patients according to the PyroScore. (B) Survival status distribution of different risk patients. (C) Heatmap showing expression levels of 8 PRGs among two risk groups. (D) Overall survival difference among the two risk groups. (E) ROC curves measure the predictive value of PyroScore in the validation cohort.

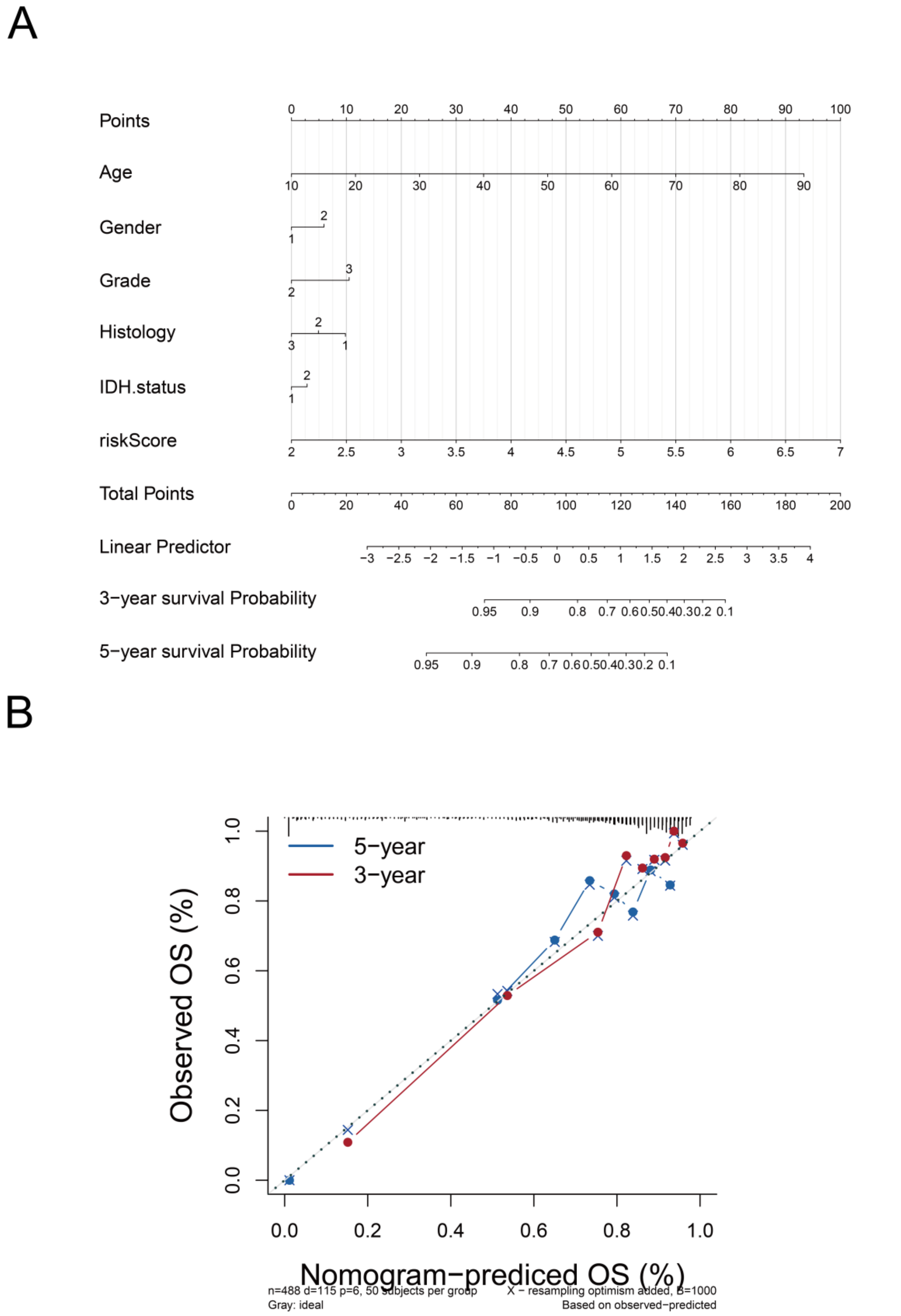

Figure S3. Combined with the PyroScore and clinical characteristics. (A) A nomogram combined with PyroScore and clinical features to quantify each patient's risk. (B) Calibration curve for the overall survival nomogram model. PyroScore is associated with immune infiltration in LGG**.**

Based on the GO analysis, the DEGs were significantly involved in T cell activation, neutrophil

degranulation, and actin-binding (Figure S4A). KEGG revealed that the DEGs were mainly enriched in the MAPK signaling pathway, salmonella infection, and proteoglycans in cancer (Figure S4B).

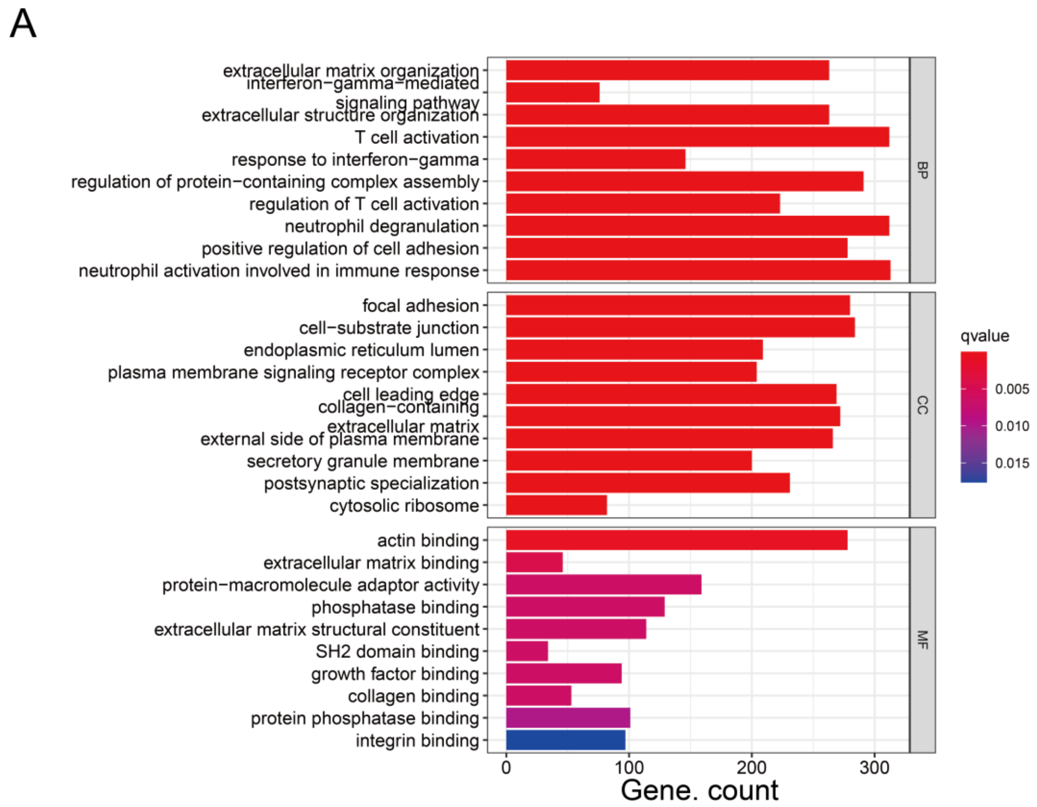

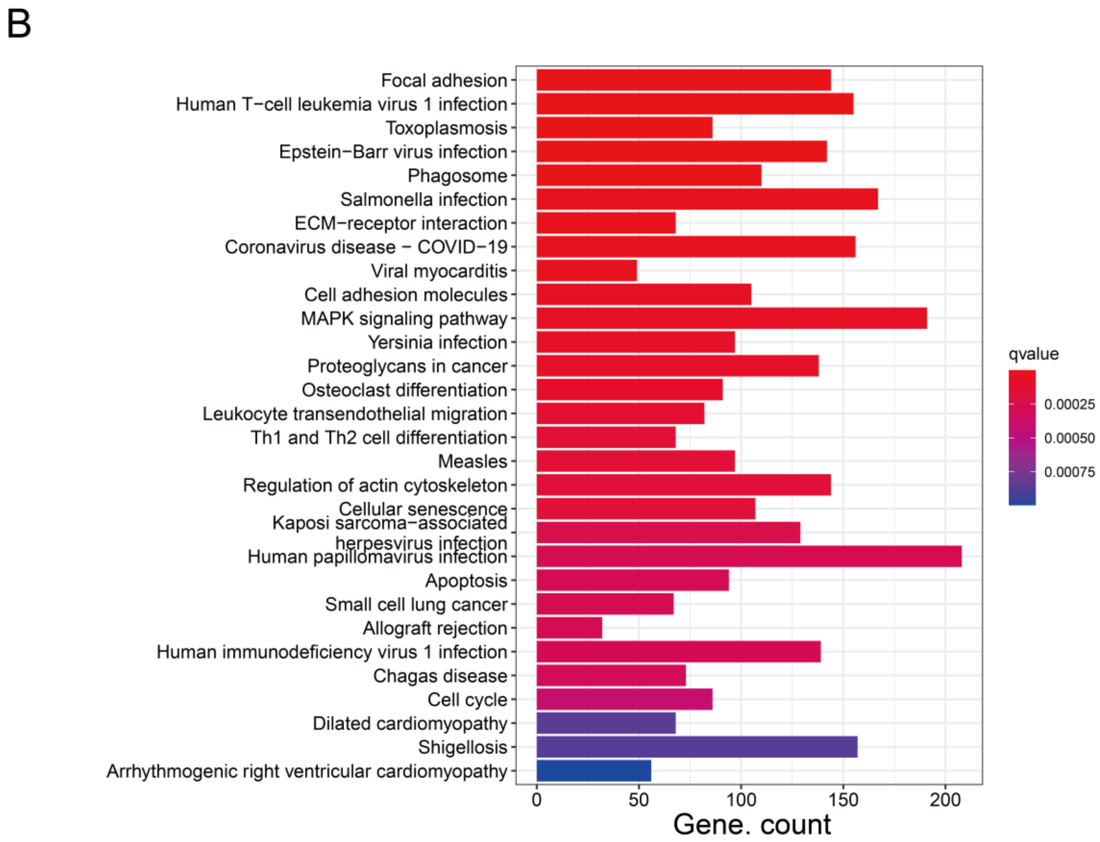

Figure S4. Functional analysis based on the DEGs between the two-risk groups. (A) gene ontology enrichment analysis. (B) Kyoto Encyclopedia of Genes and Genomes analysis. The q-value referred to the adjusted p-value; BP: biological process; CC: cellular component; MF molecular function.

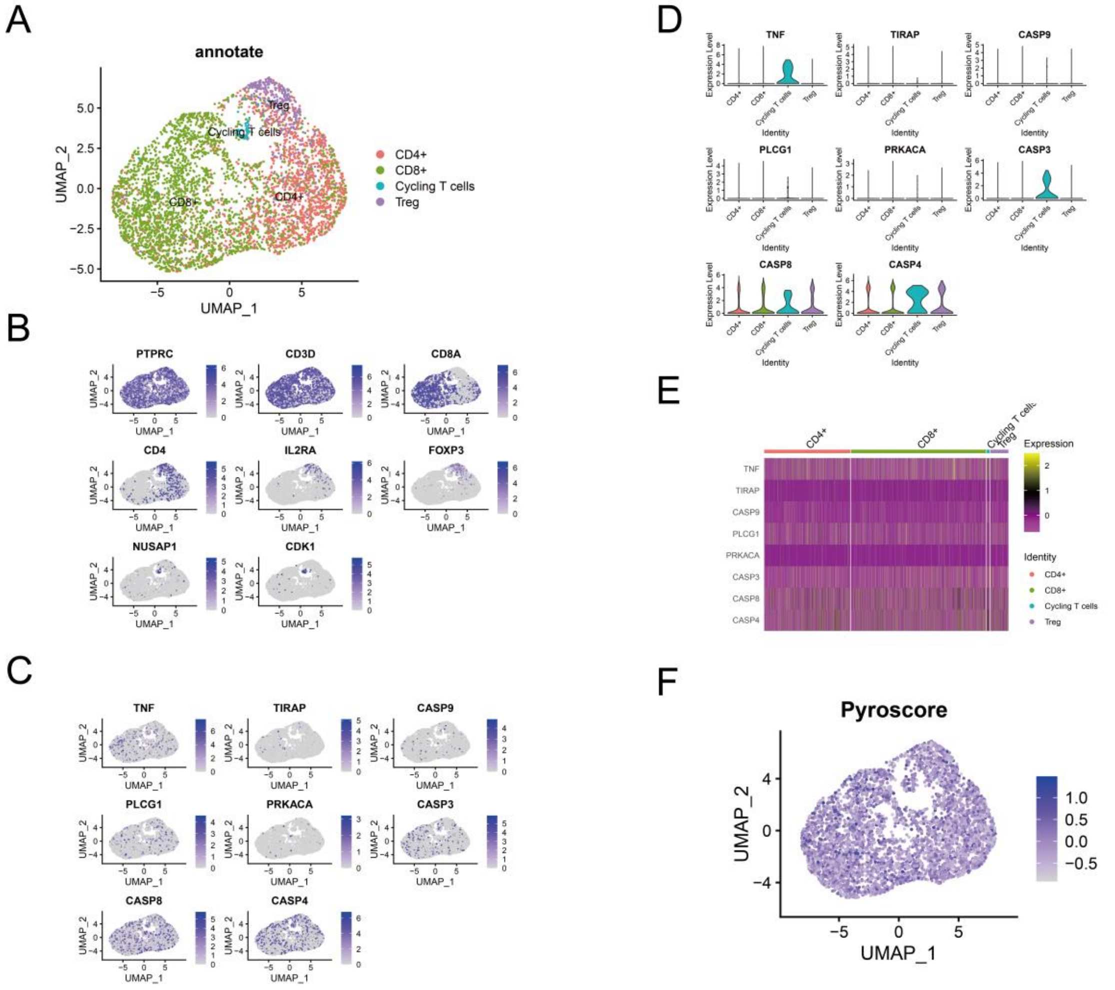

Figure S5. Single-cell analysis of PyroScore and model genes (GSE163108). (A) UMAP plots for all cells. (B) UMAP plots for marker genes. (C) UMAP plots of the eight target genes' expression: TNF, PLCG1, and CASP3/4/8, are not aggregated, although they have some basal expression. (D) Violin plots of the expression of 8 target genes in 4 T cell subtypes. (E) Heatmap of the expression of 8 target genes in 4 T cell subtypes. (F) UMAP plot for the eight PRGs: The eight target genes were used as a gene module to calculate the score using AddModuleScore.
